# Prediction of cell migration potential on human breast cancer cells treated with *Albizia lebbeck* ethanolic extract using extreme machine learning

**DOI:** 10.1038/s41598-023-49363-z

**Published:** 2023-12-14

**Authors:** Huzaifa Umar, Maryam Rabiu Aliyu, Abdullahi Garba Usman, Umar Muhammad Ghali, Sani Isah Abba, Dilber Uzun Ozsahin

**Affiliations:** 1 Near East University, Operational Research Centre in Healthcare, TRNC Mersin 10, 99138 Nicosia, Turkey; 2 Department of Energy System Engineering, Cyprus International University, Northern Cyprus via Mersin 10, 99258 Nicosia, Turkey; 3 Department of Analytical Chemistry, Faculty of Pharmacy, Near East University, TRNC, Mersin 10, 99138 Nicosia, Turkey; 4 Department of Chemistry, Faculty of Natural and Applied Sciences, Firat University, Merkezi, 23199 Elazig, Turkey; 5 Interdisciplinary Research Centre for Membranes and Water Security, King Fahd University of Petroleum and Minerals, 31261 Dhahran, Saudi Arabia; 6 Department of Medical Diagnostic Imaging, College of Health Sciences, University of Sharjah, P.O. Box 27272, Sharjah, United Arab Emirates; 7 Research Institute for Medical and Health Sciences, University of Sharjah, P.O. Box 27272, Sharjah, United Arab Emirates

**Keywords:** Biotechnology, Cancer, Drug discovery

## Abstract

Cancer is one of the major causes of death in the modern world, and the incidence varies considerably based on race, ethnicity, and region. Novel cancer treatments, such as surgery and immunotherapy, are ineffective and expensive. In this situation, ion channels responsible for cell migration have appeared to be the most promising targets for cancer treatment. This research presents findings on the organic compounds present in *Albizia lebbeck* ethanolic extracts (ALEE), as well as their impact on the anti-migratory, anti-proliferative and cytotoxic potentials on MDA-MB 231 and MCF-7 human breast cancer cell lines. In addition, artificial intelligence (AI) based models, multilayer perceptron (MLP), extreme gradient boosting (XGB), and extreme learning machine (ELM) were performed to predict in vitro cancer cell migration on both cell lines, based on our experimental data. The organic compounds composition of the ALEE was studied using gas chromatography-mass spectrometry (GC–MS) analysis. Cytotoxicity, anti-proliferations, and anti-migratory activity of the extract using Tryphan Blue, MTT, and Wound Heal assay, respectively. Among the various concentrations (2.5–200 μg/mL) of the ALEE that were used in our study, 2.5–10 μg/mL revealed anti-migratory potential with increased concentrations, and they did not show any effect on the proliferation of the cells (P < 0.05; n ≥ 3). Furthermore, the three data-driven models, Multi-layer perceptron (MLP), Extreme gradient boosting (XGB), and Extreme learning machine (ELM), predict the potential migration ability of the extract on the treated cells based on our experimental data. Overall, the concentrations of the plant extract that do not affect the proliferation of the type cells used demonstrated promising effects in reducing cell migration. XGB outperformed the MLP and ELM models and increased their performance efficiency by up to 3% and 1% for MCF and 1% and 2% for MDA-MB231, respectively, in the testing phase.

## Introduction

Cancer is a primary cause of death in the modern world, and the incidence varies considerably based on race, ethnicity, and region. Several studies revealed metastases responsible for 90% of cancer deaths^1–4^. Breast cancer (BCa) is among the leading causes of death in the United States and other parts of the world^5^. A recent report by the WHO (2018) shows that breast cancer accounts for about 0.627 million women’s mortality. However, BCa patients’ mortality rate increases from systematic metastases to distant organs^5^.

Therefore, there is a need for effective chemo-preventive agents with fewer side effects to suppress tumour metastasis. BCa metastasis involves complex processes established by various pathways and factors. It starts with cell motility in the primary affected site to other distant tissue, blood, or lymph vessels^6,7^. Metastasis involves exiting cancerous cells from the primary site to invade various organs and tissues, facilitating cellular migration, invasion, and adhesion mediated by the Ca^2+^ signalling process^8^. Metastasis maintained primary tumour-differentiated characteristics such as cell-to-cell contact, signalling, and behaviour. Transforming Growth Factor (TGF) and Epidermal Growth Factor (EGF) signalling can trigger breast cancer tumour motility^9,10^. Consequently, understanding the mechanisms behind BCa metastasis and combatting it is paramount in defeating the war against BCa^11^.

Therapies and drugs are available to cure cancer, but still, there is a need for effective therapies and targeted plant-based medications with fewer side effects^12^. Various chemotherapeutic treatments with paclitaxel and anthracyclines might induce apoptosis, inhibit cell proliferation, and affect cellular activity^13^. Natural products such as plants, marine organisms, and microorganisms have revealed potential in cancer treatments^14^. Scientists continuously search for anticancer drugs that will have low toxicity and side effects but high efficacy. One such approach is targeting apoptotic cells, usually targeted as a step during therapy. Novel cancer treatments such as surgery and immunotherapy are not obligatory and quite expensive. In this situation, ion channels responsible for cell migration (metastasis) have appeared to be the most promising targets in the last two decades^14,15^.

*Albizia lebbeck* leaves are natural products rich in flavonoids and demonstrate antitumor activity against HepG2 hepatoma cells. Zinc oxide nanoparticles synthesized using plant stem bark revealed cytotoxic activity against highly and weakly metastatic human BCa cells^15,16^. Quercetin is one such flavonoid founds in *Albizia lebbeck* that is not toxic, and it demonstrates many biological actions, including anticancer, anti-inflammatory, and antioxidant activity^4,18,19^. Quercetin has also induced cell circle arrest and apoptosis through signalling pathways and FOXO3a modification in breast cancer cells^20^.

Modelling cancer cell migration in vitro has been a venerable challenge due to various complexities in cellular and molecular regulatory mechanisms in the system^21^. Conventionally, studies report the employment of rule-based greedy algorithms within agent-based modelling (ABM) in the modelling of cell migration^22,23^. Zhang et al. describe metastasis as the primary cause of death in many breast cancer patients. The cancer cell migration behaviour was analysed computationally by applying high-content imaging and microfluidic single-cell migration. Random forest decision and artificial neural network (ANN) were employed in the study. The results indicated higher accuracy regarding the prediction of cell movement^24^. Ravdin et al. described the application of artificial intelligence for predicting clinical outcomes of node-positive breast cancer patients. The status of the hormone receptor, tumour size, age of the patient, as well as relapse status were used as the input variables. The results prove a satisfactory neural network in predicting cancer status in patients^25^. Furthermore, Jerez et al. employed various machine learning and statistical methods that can be used to simulate the recurrence of breast cancer patients. The results prove the reliability of the machine learning data algorithms over the classical statistical processes in the simulation of breast cancer outcomes^26^. Based on the studies mentioned above from the technical literature, it can be observed that the applications of artificial intelligence are of paramount importance, which shows reliable and satisfactory results over the classical statistical methods. Moreover, since the developments of artificial intelligence-based models, this is the first work conducted in the technical literature depicting the application of these kinds of novel artificial intelligence models for the prediction of lateral motility in human BCa cells. However, we employed non-linear models in our study due to their flexibility, predictability, precision,and interpretability.

In this present study, the organic compounds present in *Albizia lebbeck* ethanolic extracts (ALEE), as well as their impact on anti-migratory, antiproliferative and cytotoxic potential on MDA-MB 231 and MCF-7 human BCa cell lines. In addition, the artificial intelligence (AI) based models, multilayer perceptron (MLP), extreme gradient boosting (XGB), and extreme learning machine (ELM), were performed to predict in vitro cancer cells migration on MDA-MB 231 and MCF-7cells, based on our experimental data.

## Materials and methods

### Plant material

Fresh stem barks of *A. lebbeck* were collected during the rainy season (April to October) from northern Nigeria, a town called Tabuli, part of Gaya Local Government, Kano State, during their flowering stage and dried at room temperature. The *A. lebbeck* stem bark collection follows all the applicable international standards, guidelines, and laws. The plant specimen was authenticated by Dr. Bala Sidi Aliyu, and deposited with voucher specimen number BUKHAN187 at the herbarium Plant Biology Department, Faculty of Science, Bayero University Kano.

### Sample preparation

Dried *Albizia lebbeck* stem barks were pulverised to clear powder and subjected to flask extraction using 99.9% methanol as extraction solvent. Powdered *A. lebbeck* stem bark (50 g) was soaked in an Erlenmeyer flask containing methanol (500 mL) and placed under continual shaking for 48 at room temperature^27^. Whatman filter paper No.1 was used to filter the extract and concentrate it under reduced pressure using a Rotary evaporator. The concentrated extract was dried completely at 40 °C in an oven and stored at 4 °C before the analysis.

### Phytochemical analysis of the extract

The ALEE extracts were analysed for their total flavonoid (TFC) and total phenolic content (TPC) using standard spectrophotometric methods^28,29^. To determine TFC determination, ALEE (1 mg/mL) was mixed with NaNO2 solution (5%), 10% AlCl3, and 1 M NaOH, and absorbance was measured at 510 nm. Folin-Ciocalteu reagent was added to ALEE (10:1) for TPC, followed by incubation with Na_2_CO_3_ (7.5%) and absorbance measurement at 760 nm. Results are presented as mg quercetin equivalent (QE)/g dry extract and gallic acid equivalents (µg GAEs/g dry extract).

We utilised gas chromatography-mass spectrometry (GC–MS) to analyse the organic composition of ALEE. We first created a crude extract in ethanol (1 mg/mL) and filtered it via a 0.22 µm syringe filter. Then, we injected it into a Shimadzu GC–MS–QP2010 plus analyser with helium as the carrier gas at a steady flow rate of 1 mL/min. The oven temperature was set at 50 °C for 2 min and gradually increased by 7 °C/min. We assessed the mass spectra at a scanning interval of 0.5 s, with a complete scan range from 25 to 1000 m/z, employing a Quadrupole mass detector. Ultimately, we identified the existing compounds by scrutinising the spectrum via the WILLEY7 MS library.

### Cell models and culture conditions

MDA-MB 231 (strongly metastatic) and MCF-7 (weakly metastatic) BCa cell lines were obtained as a gift from Imperial College London (UK) and stored at the Biotechnology Research Centre (BCR) of Cyprus International University. The BCR ethical committee (BRCEC2011-01) approved using these cell lines in our study. We cultured the cells in Dulbecco's Modified Eagle's Medium (DMEM) (Gibco by Life Technology USA), supplemented with 2 mM L-glutamine, penicillin, and 10% fetal bovine serum (FBS), and maintained them in a sterile incubator at 37 °C and 5% CO_2_.

### Toxicity and proliferation assay

We conducted a tryphan blue dye exclusion assay, following the guidelines provided by Fraser et al.^31^, to measure the level of cytotoxicity in BCa cells. We administered various doses, 0, 2.5, 10, 25, 50, 100 and 200 μg/mL, to the cells and observed them for 24, 48, and 72 h. After this period, we replaced the medium with a diluted tryphan blue solution, formulated by mixing 0.25 ml of the dye with 0.8 ml of medium. This assay accurately determined the extent of cytotoxicity present in the cells. Data are presented as averages of 3 × 30 measurements.

The proliferation of MDA-MB 231 (strongly metastatic) and MCF-7 (weakly metastatic) BCa cells treated with ALEE extracts were assessed using MTT (3-[4,5-dimethylthiazol-2-yl]-2,5-diphenyltetrazolium bromide) reagent Sigma-Alderich) as described by Fraser et al. (1990) with some adjustments. BCa cells (3** × **10^4^ cells/mL) cultured in tissue plates (12-well) were treated with 10, 5, 2.5, and 0 μg/mL of ALEE extracts and incubated for 24, 48, and 72 h. Treatments and culture medium (DMEM) were replaced every 24 h. Microplate Reader (ELX 800™) was used to measure the absorbance of the treated cell and control at 570 nm. All the experiment was performed at least thrice in triplicates (*n* ≥ 3).

### Wound heal assay

A wound heal assay was carried out to evaluate the anti-metastatic potential of ALEE extracts against highly metastatic (MDA-MB 231) and weakly metastatic (MCF-7) cells using the method of Fraser et al*.* with some modifications. Cells were plated in 35 mm culture dishes, and parallel and intersecting lines were drawn on the culture dishes^31^. Briefly, 1 × 10^6^/mL and 5 × 10^5^/mL cells per dish of MCF-7 and MDA-MB 231, respectively, were plated on 35 mm culture dishes, and three scratch lines were made using pipette tips (200 μL) after the cell settled. The initial and subsequent wounds causedwere captured using a camera (Leica, Germany) attached to an inverted microscope at × 100 magnification, and image processing software (ImageJ) was used to analyse the recovery wound area (cell migration) by migrating cells using Eq. (1).

1 $$\mathrm{Mo I}=1-(\frac{ {\text{Wt}}}{\mathrm{ W}0})$$

Mo I, motility index; W_t_, the wound width at 24 or 48 h; W_0,_ initial wound width at 0 h.

### Modelling approach

**Figure 1:**
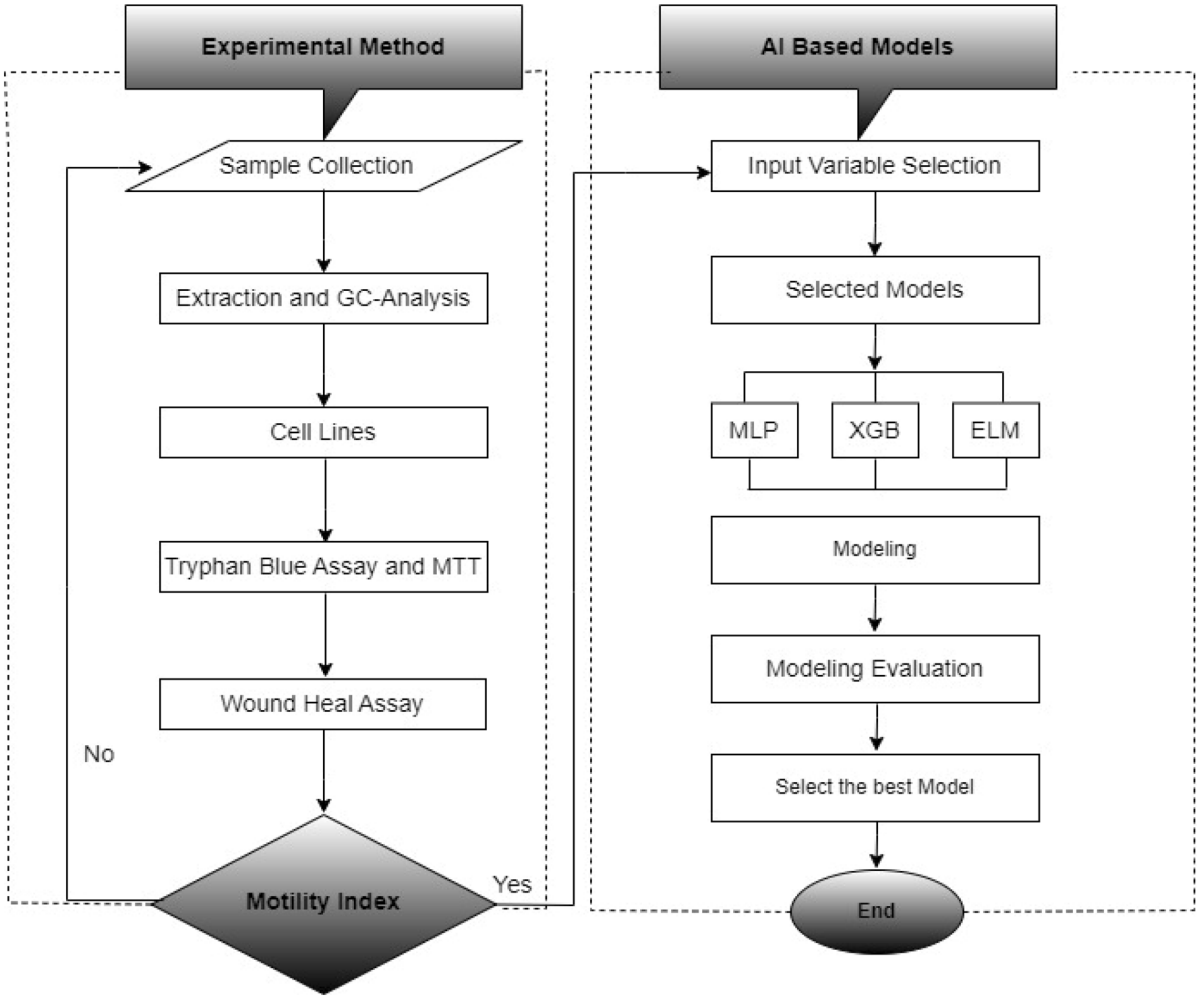
Proposed flowchart of experimental data-driven methods.

The study of the science of data is critical in any driven-model data-driven model. The accuracy of the data was tested using XGB, ELM, and MLP algorithms with MATLAB (R2021a). In this work, various models were proposed for the in vitro cancer metastasis prediction in MDA-MB 231 and MCF-7 cells, respectively. The data was collected from our experimental data set (n ≥ 80) to reveal the accuracy of the algorithms. In this way, two parameters were used as input variables, i.e. the motility index on the cells and the concentration of the extract, respectively. The two parameters we considered in modelling were the concentration of the extract and the motility index, although other parameters can be utilized for the same purpose. The models used have a learning algorithm with a single layer, and a fast learning rate and both the hidden biases and input layers which process and distribute data respectively, in the network are chosen randomly. However, other variables can also be used in the simulation of in vitro cancer metastasis prediction in both cell lines. In addition, models provide details on the effectiveness of the treatment, and choosing a single model that can perform best in most circumstances is difficult for the predictors, but applying various ensemble models can reveal the best models that will fit the data. Determination of cell migration potentials in breast cancer cells treated with ALEE extract using the motility index on the cells and the extract concentration as the input parameters were the main objectives of our proposed method. The proposed flowchart of the models is shown in Fig. 1.

### Extreme gradient boosting (XGB)

The XGB algorithm is a commonly used model that is highly efficient with high reproducibility in analysing and modelling data using various inputs and outputs. The method was first introduced and improved by Friedman et al*.*^32^, and it plays an essential role in the classification and regression of data. Its application in extreme learning techniques is well-known and the technique^33^. The technique uses a precise setup of up best complex decision tree algorithm to reveal good performance and speed faster than the standard gradient algorithm^34^. XGB is a machine learning ensemble technique that works similarly to Random Forest and is recognised by its classification and regression trees (CART) set. The model utilizes parallel processing to enhance learning speed, balance between variance and bias, and minimize the risk of overfitting. Furthermore, it is not the same with the decision tree (DT), whereby every leave carries an actual score, which aids in enriching those interpretations which cannot be defined using the DT. Algorithms have been used in modelling and predicting data, and it has shown promising results. Due to this ensemble technique's wide application and excellent features, we use it to model and predict the anti-migratory potential of the cells. Given that CART $[(xi, yi)\dots ..{\text{T}}K(xi, yi)]$ is the training data set of the treated cells motility index represented as x_i_ to predict outcomes y_i_ and determined using K classification, as shown in Eq. (2)^35^:

2 $$\widehat{y}= \sum_{k=1}^{K}{f}_{k}\left({x}_{i}\right), {f}_{k}\in F$$

where ${f}_{k}$ represents independent tree structure with cells motility index scores, and *F* denotes the space of all CART. Optimisation of the objective is given by Eq. (3)^35^:

3 $$obj\left(\theta \right)= \sum_{i=1}^{n}l({y}_{i}, {\widehat{y}}_{i})+\sum_{i=1}^{t}\Omega ({f}_{i})$$

The loss function is denoted $l$ which estimates the difference between target ${y}_{i}$ and predicted ${\widehat{y}}_{i}$. The regularization function that penalises the model to avoid over-fitting is denoted as $\Omega ,$ and ${f}_{i}$ represents the simultaneous training loss function. Furthermore, the prediction value for $t$ at step ${\widehat{y}}_{i}^{t}$^35^:

Prediction $\widehat{y}$ at the t step can be expressed as

4 $${\widehat{y}}_{i}^{t}=\sum_{k=1}^{t}{f}_{k}\left({x}_{i}\right)={\widehat{y}}_{i}^{t-1}+{f}_{t} ({x}_{i})$$

Substituting the predicted value in Eq. (4). Equation (3) can be expressed as^36^:

5 $${obj}^{t}= \sum_{i=1}^{n}({{y}_{i}-({\widehat{y}}_{i}^{t-1}+{f}_{t} \left({x}_{i}\right)))}^{2}+\sum_{i=1}^{t}\Omega ({f}_{i})$$

It can also be expressed as

6 $${obj}^{t}= \sum_{i=1}^{n}{[ 2\left( {\widehat{y}}_{i}^{t-1}-{y}_{i}\right){f}_{t} \left({x}_{i}\right) +{f}_{t} \left({x}_{i}\right)}^{2}+\Omega ({f}_{t})+constant$$

Looking at Taylor’s expansion due to loss of function, it can be expressed in Eq. (7)^36^:

7 $${obj}^{t}= \sum_{i=1}^{n}{[ l\left({y}_{i},{\widehat{y}}_{i}^{t-1}\right)+{g}_{i} {f}_{t} \left({x}_{i}\right) +{\frac{1}{2}{h}_{i} f}_{t} \left({x}_{i}\right)}^{2}+\Omega ({f}_{t})+constant$$

where ${g}_{i}= {\partial }_{{\widehat{y}}_{i}^{t-1}}{l(y}_{i}-{\widehat{y}}_{i}^{t-1})$, and ${h}_{i}= {\partial }_{{\widehat{y}}_{i}^{t-1}}^{2}{l(y}_{i}-{\widehat{y}}_{i}^{t-1})$. Which was described by ${f}_{t}\left(x\right)= {w}_{q(x)},$ and the normalised function is expressed as

8 $$\Omega \left(f\right)= \gamma T+\frac{1}{2}\lambda \sum_{j=1}^{T}{w}_{j}^{2}$$

where $T$ represent the total number of trees, and the objective function can rewritten as

9 $${obj}^{t}\approx \sum_{i=1}^{n}[{g}_{i} {w}_{q\left({x}_{i}\right)}+ \frac{1}{2}{h}_{i}{w}_{q\left({x}_{i}\right)}^{2}]+\gamma T+\frac{1}{2}\lambda \sum_{j=1}^{T}{w}_{j}^{2}=\sum_{j=1}^{T}[\left(\sum_{i\in {I}_{i}}{g}_{i}\right){w}_{j}+ \frac{1}{2}\left(\sum_{i\in {I}_{i}}{h}_{i}+\lambda \right){w}_{j}^{2}+ \gamma T$$

where ${I}_{i}=\{\left.i\right| q\left({x}_{i}\right)=j\}$ refers to the ${j}^{th}$ leaf data index. ${G}_{j}=\sum_{i\in {I}_{i}}{g}_{i}$ and ${H}_{j}=\sum_{i\in {I}_{i}}{h}_{i}$, the objective function can be written as

10 $${obj}^{t}= \sum_{j=1}^{T}[{G}_{j}{w}_{j}+\frac{1}{2}\left({H}_{j}+ \lambda \right){w}_{j}^{2}+\gamma T$$

Performance for $q(x)$ can be achieved using the objective function and ${w}_{j,}$ as you can see in Eqs. (11) and (12).

11 $${w}_{j}^{*}= -\frac{{G}_{j}}{{H}_{j}+ \lambda }$$

12 $${obj}^{*}= - \frac{1}{2} \sum_{j=1}^{T}\frac{{G}_{j}}{{H}_{j}+ \lambda }+\gamma T$$

In addition, Eq. (13) is for leaf node score during splitting, L and R are the left and right scores, and the regularisation of the additional leaf is denoted as $\gamma$.

13 $$Gain= \frac{1}{2}\left[\frac{{G}_{L}^{2}}{{H}_{L}+\lambda }+ \frac{{G}_{R}^{2}}{{H}_{R}+\lambda }-\frac{{({G}_{L}+{G}_{R})}^{2}}{{H}_{L}+ {H}_{R}+ \lambda }\right]-\gamma$$

### Extreme learning machine

The ELM model is a novel learning algorithm with a single hidden layer that works similarly to a feed-forward neural network (FNN) due to its approximation potential. And it was first introduced by Huang et al.^37^. Issues such as slower training speed and over-fitting with FNN have been addressed analytically by ELM through inversion and matrix multiplication^38^. The structure of this model contains only one layer and hidden nodes, which result in the model not requiring a learning process to calculate its parameters, and hence, it remains constant during both the training and predicting phases. In addition, ELM hidden biases and input layer are chosen randomly, and the Moore–Penrose generalised inverse function determines the output layer. The ELM revealed precision due to its robustness when applied to hydrological.

Modelling^39^.

The ELM was expressed by training dataset $\{\left({x}_{1}, {y}_{1}\right), \dots , \left({x}_{t}, {y}_{t}\right)\}$. Overall, the input are represented as ${x}_{1}, {x}_{2}, \dots , {x}_{t}$ and the output as ${y}_{1}, {y}_{2}, \dots , {y}_{t}$.

The training dataset $N$ ($t = 1, 2, \dots , N$) where ${x}_{t} \in {\mathbb{R}}^{d}$ and ${y}_{t}\in {\mathbb{R}}$, with $H$ hidden nodes, is given by^37^ as in Eq. (14):

14 $$\sum_{i=1}^{H}{B}_{i}{g}_{i}\left({\alpha }_{i}.{x}_{t}+{\beta }_{i}\right)= {z}_{t},$$

Equation (14), $i$ represents index of the hidden layer node, ${\beta }_{i}$ and ${\alpha }_{i}$ denote the bias and weight of the random layers, and $d$ is the number of inputs. Furthermore, the predicted weight of the output layer, model output and hidden layer neurons activation function are $B \in {\mathbb{R}}^{H}$, $Z({z}_{t}\in {\mathbb{R}})$ and $G\left(\alpha ,\beta , x\right)$ respectively. The best activation function is found to be the sigMoId function^40^ as follows:

15 $$G\left(x\right)= \frac{1}{1+exp(-x)},$$

In addition, the output layer utilizes a linear activation function, which is shown in the following equation:

16 $$\sum_{t=1}^{N}\Vert {z}_{t}-{y}_{t}\Vert =0,$$

The value of $B$ is calculated using the system of linear equations as expressed in Eq. (17) and G in Eq. (18)

17 $$Y=GB$$

18 $$G\left(\alpha ,\beta , x\right)= \left[\begin{array}{c}g({x}_{1})\\ \vdots \\ g({x}_{N})\end{array}\right]={ \left[\begin{array}{ccc}{g}_{1}({\alpha }_{1}.{x}_{1}+{\beta }_{1})& \cdots & {g}_{L}({w}_{H}.{x}_{1}+{\beta }_{H})\\ \vdots & \cdots & \vdots \\ {g}_{1}({\alpha }_{N}.{x}_{N}+{\beta }_{1})& \cdots & {g}_{L}({w}_{H}.{x}_{N}+{\beta }_{F})\end{array}\right]}_{N \times H}$$

B is calculated in Eq. (19), and Y in Eq. (20).

19 $$B={\left[\begin{array}{c}{B}_{1}^{T}\\ \vdots \\ {B}_{H}^{T}\end{array}\right]}_{H \times 1}$$

20 $$Y={\left[\begin{array}{c}{y}_{1}^{T}\\ \vdots \\ {y}_{N}^{T}\end{array}\right]}_{N \times 1}$$

G is for the hidden layer. $\widehat{B}$ was calculated using “Moore–Penrose inverse function + by inverting the hidden-layer matrix” (see Eq. 21).

21 $$\widehat{B}={G}^{+}Y$$

Overall, estimated $\widehat{y,}$ which denotes the predicted MoI of the cells whic,h can achieved using Eq. (22).

22 $$\widehat{y}= \sum_{i=1}^{H}{\widehat{B}}_{i}{g}_{i}\left({\alpha }_{i}.{x}_{t}+{\beta }_{i}\right)$$

### Multilayer perceptron

**Figure 2:**
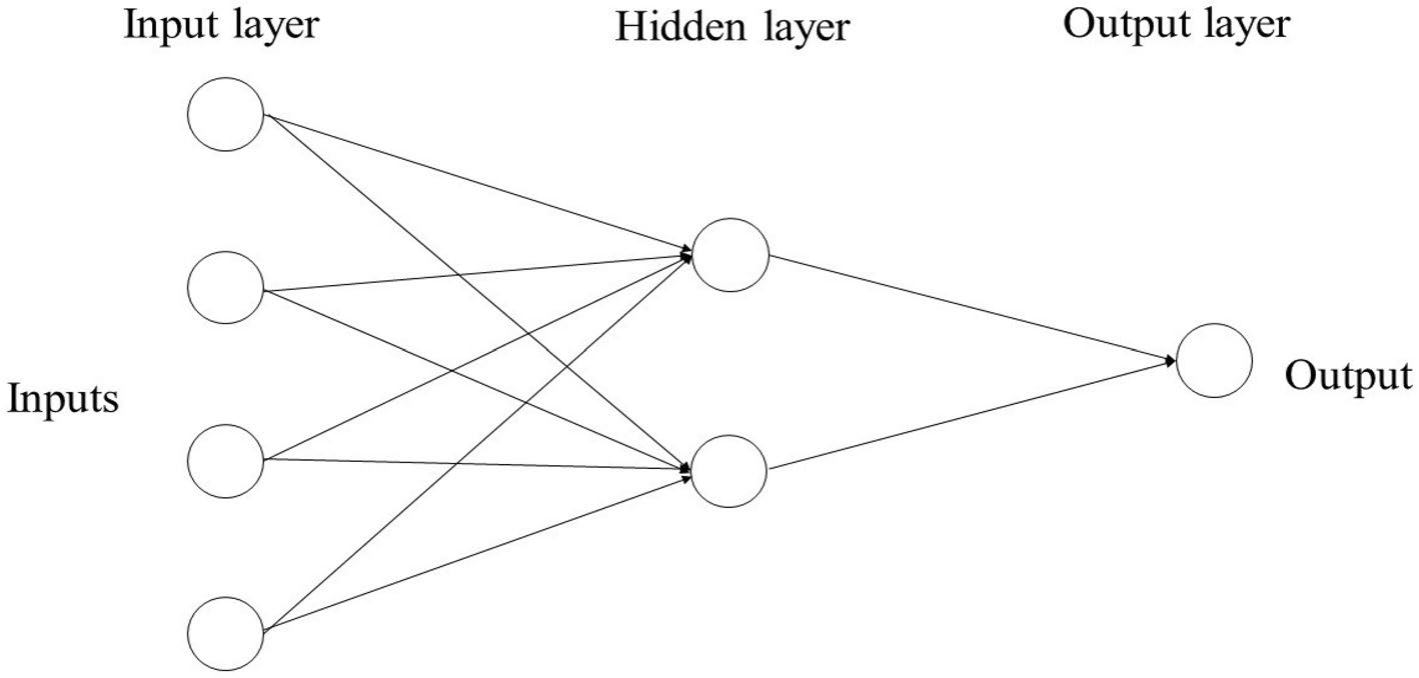
Schematic diagram of MLP network structure.

MLP, as one of the commonly applied Artificial neural networks (ANNs) composed of information processing units and an advanced simulation tool, motivated and mimicked the biological neurons. In this way, ANN, just like the human central nervous system (CNS), can solve complex problems with a non-linear and linear behaviour by combining features such as parallel processing, generalisation, learning power and decision making^41^. The general architecture of ANN consists of 3 layers with individual and different tasks: the input layer, which distributes the data in the network; the hidden layers, which process the information and the outputs, which, in addition to processing each input vector, show its work. The neurons are regarded as the smallest unit that processes the networks. The basic characteristics of MLP include using interactive connections between the neurons without advanced mathematical design to complete the information processing. Furthermore, MLP comprises input, one or more hidden and output layers in its architecture, similar to the ANN (Fig. 2)^40^.

### Performance objectives

To evaluate the performance efficiency of the artificial intelligence-based models used in the current study; two different metrics, where; Nash–Sutcliffe coefficient (NS) was used for understanding the fitness between the experimental and predicted values, while Root mean square error (RMSE) was used in determining the errors depicted by each model.

Hence, the Root mean square error (RMSE) was expressed as:

23 $$RMSE=\sqrt{\frac{1}{N}} \sum_{j=1}^{N}{\left({(Y)}_{obs,j}-{(Y)}_{com,j}\right)}^{2}$$

Nash–Sutcliffe coefficient (NS), expressed as:

24 $${\text{NS}}=1-\left[\frac{{\sum }_{i=1}^{N}{\left({Q}_{obs,i}-{Q}_{sim,i}\right)}^{2}}{{\sum }_{i=1}^{N}{\left({Q}_{obs,i}-{\overline{Q} }_{obs,i}\right)}^{2}}\right]\infty \le NS\le 1$$

## Result and discussion

### Experimental results

The study found that the ALEE contained TFC and TPC at levels of 2022.80 ± 17.83 QE µg/g and 6556.49 ± 22.52 GAE µg/g, respectively. Studies have shown that TPC is highly efficient in scavenging different oxidizing molecules, including free radicals produced during lipid peroxidation^42^. Moreover, research has revealed that flavonoids, present in various structures of phenolic compounds, possess medicinal properties. These compounds can be found in sources such as flowers, leaves, stem bark, roots, fruits and tea^43,44^.

**Figure 3:**
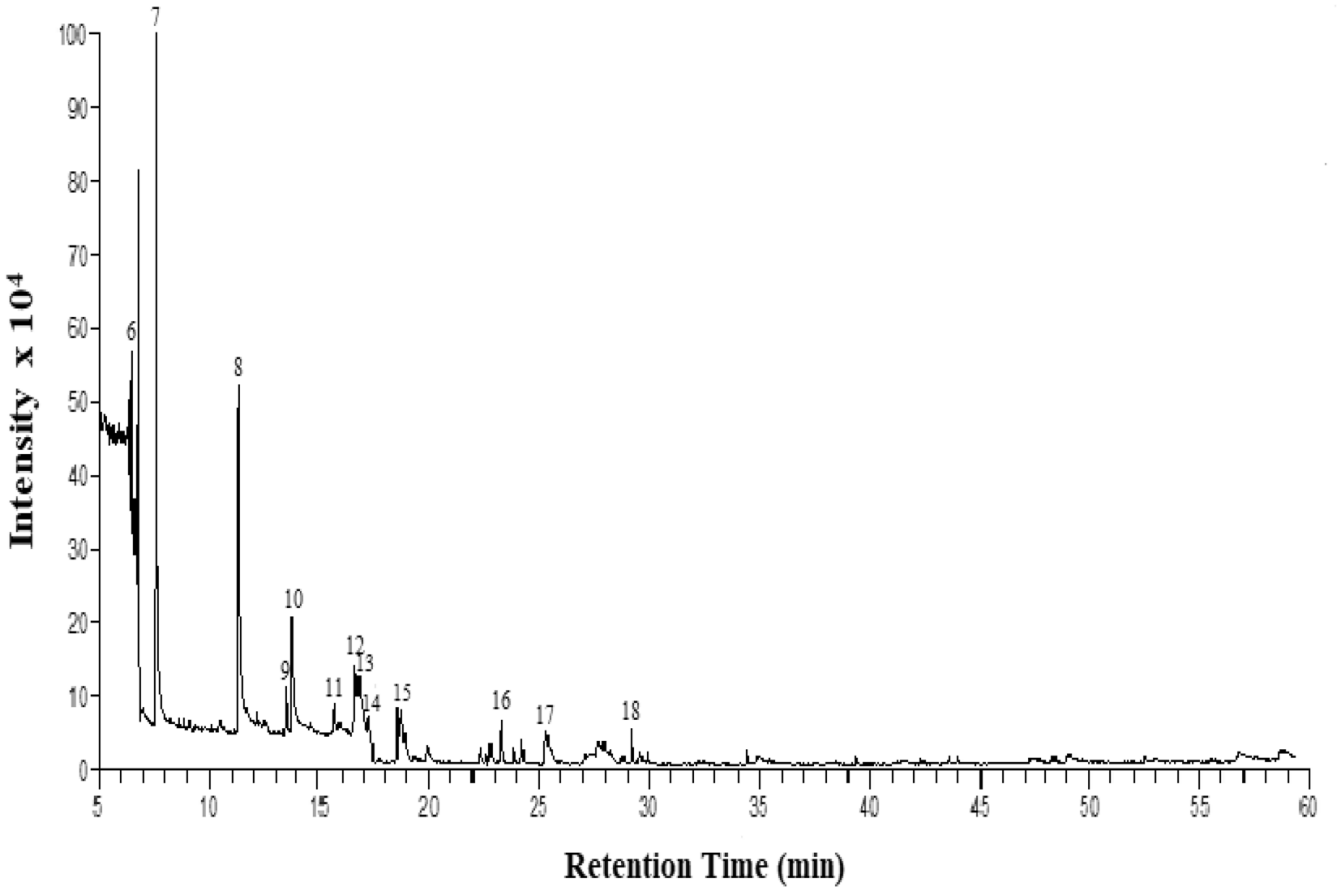
GC–MS chromatogram of ALEE bioactive compounds. The numbered peaks correspond to the numbers and molecules in Table 1.

The compounds found in ALEE are listed in Table 1; their corresponding chromatogram peaks are shown in Fig. 3. We identified several significant compounds in our extract that have biological potential; some of them are Ethanol (88.55%), Silicic acid, diethyl bis(trimethylsilyl) ester (3.18%), 1-cyano-5-benzoyloxy-á-d-ribofuranose (1.67%), 1-(2-trimethylsiloxy-1,1-dideuteriovinyl)-4-trimethyl siloxy-benzene (1.21%), Disiloxane, 1,3-diethoxy-1,1,3,3-tetramethyl- (1.43%), 1,2-Dihydro-1,4-diphenylphthalazine (0.75%), 3-(4'-Methoxyphenyl)-1-acetyl-2-phenylindolizine (0.52%), 4H-3-(p-methylamino)1-benzothiopyran-4-one 1-oxide (0.40%). The high percentage of ethanol might be from the extraction solvent, indicating that it is not a suitable solvent for *A. lebbeck* extraction. Furthermore, the extract's anti-proliferative and anti-migratory potential might result from the phytochemicals present in the extract, and ALEE could be a good cause that will prevent the metastasis of breast cancer.

**Table 1:** Bioactive compounds identified from ALEE by GC–MS analysis and their biological activities.

S/n	RT	Peak area	Area %	Compound detected	Biological activity
1	2.00	2,506,581,156	88.55	Ethanol (CAS)	Anti-microbial^45^
2	3.08	2,369,689	0.08	(S)-(E)-(−)-4-Acetoxy-1-phenyl-2-dodecen-1-one	Anti-cancer, DNA-binding and anti-microbial^46^
3	3.77	13,527,363	0.48	Cyclotrisiloxane, hexamethyl-(CAS)	Anti-cancer, anti-oxidant and anti-microbial^47^
4	3.98	21,342,385	0.75	1,2-Dihydro-1,4-diphenylphthalazine	Anticancer^46^
5	6.46	90,085,996	3.18	Silicic acid, diethyl bis(trimethylsilyl) ester	-
6	6.74	34,182,822	1.21	1-(2-trimethylsiloxy-1,1-dideuteriovinyl)-4-trimethylsiloxy-benzene	Anti-microbial^45^
7	7.60	47,188,509	1.67	1-cyano-5-benzoyloxy-á-d-ribofuranose	Anticancer^46^
8	11.33	40,503,790	1.43	Disiloxane, 1,3-diethoxy-1,1,3,3-tetramethyl- (CAS)	Anti-inflammatory, anti-bacterial^48^
9	13.47	3,392,203	0.12	(S)-(E)-(−)-4-Acetoxy-1-phenyl-2-dodecen-1-one	Anti-cancer, DNA-binding and anti-microbial^46^
10	13.76	15,872,886	0.56	Silicic acid, diethyl bis(trimethylsilyl) ester	-
11	15.69	5,409,162	0.19	Silicic acid, diethyl bis(trimethylsilyl) ester	-
12	16.67	8,613,439	0.30	2-(5-acetyl-2-thienyl)-1,4-naphthoquinone	Anti-microbial, anti-tumor and antiproliferative^46^
13	16.84	11,375,011	0.40	4H-3-(p-methylanilino)1-benzothiopyran-4-one 1-oxide	Anti-cancer^46^
14	17.29	3,343,231	0.12	Heptasiloxane,1,1,3,3,5,5,7,7,9,9,11,11,13,13-tetradecamethyl-	Anti-oxidant and anti-inflammatory^48^
15	18.70	14,832,408	0.52	3-(4'-Methoxyphenyl)-1-acetyl-2-phenylindolizine	-
16	23.31	3,451,675	0.12	Cyclooctasiloxane, hexadecamethyl-	Anti-cancer, anti-oxidant and anti-microbial^47^
17	25.33	6,562,803	0.23	4-Ethylbenzoic acid, 2-butyl ester	Anti-cancer and Anti-mibroabial^47^
18	29.18	2,201,203	0.08	Cyclononasiloxane, octadecamethyl-	Anti-cancer, anti-oxidant and anti-microbial^48^
18	29.18	2,201,203	0.08	Cyclononasiloxane, octadecamethyl-	Anti-cancer, anti-oxidant and anti-microbial^46^

**Figure 4:**
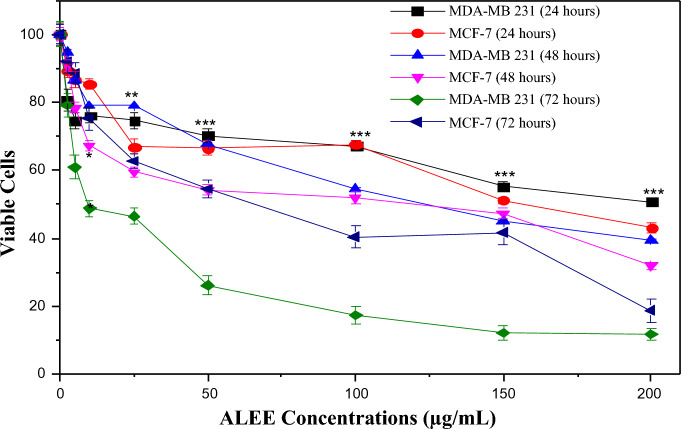
Cytotoxic Effect of various concentrations of ALEE MDA-MB 231 and MCF-7 breast cancer cell lines carried out using tryphan blue exclusion assay. The observations were made after 24, 48 and 72 h’ incubation period. The findings are presented as mean ± SEM of at least replicates experiments (*n* ≥ 3) and analysed using one-way ANOVA followed by Newman–Keuls post hoc analysis. (*) P < 0.05; (**) P < 0.01 and (***) P < 0.0001.

**Figure 5:**
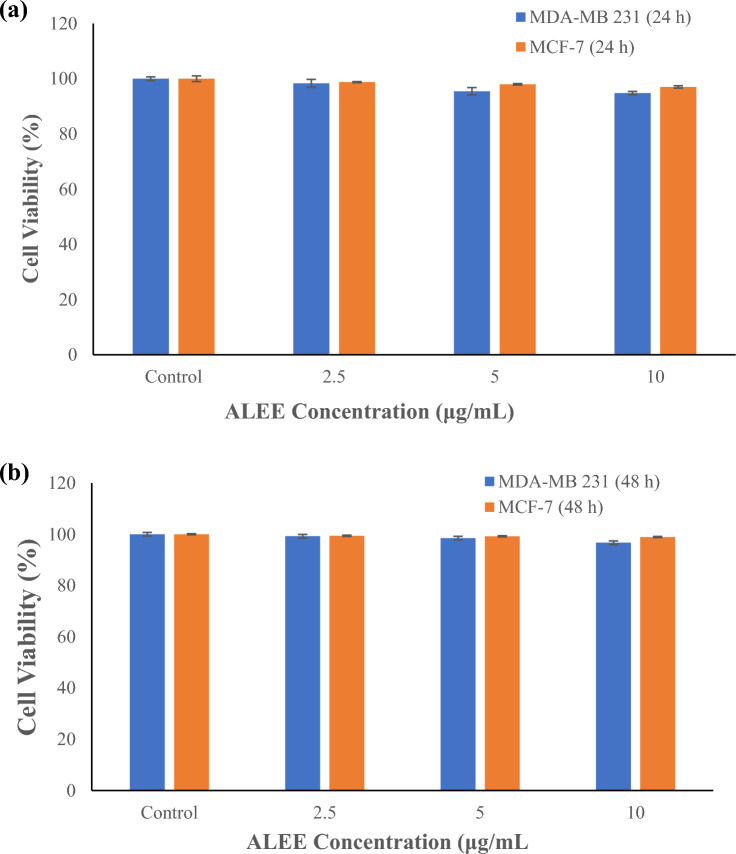
Effect of ALEE on proliferation of MDA-MB 231 and MCF-7. MDA-MB 231 and MCF-7 were treated with various concentrations of ALEE for (**a**) 24 h and (**b**) 48 h carried out using MTT Assay. Data are presented as mean ± SEM of at least replicates experiments (*n* ≥ 3), and analysed using one-way ANOVA followed by Newman-Keuls post hoc analysis. (*) P < 0.05; (**) P < 0.01 and (***) P < 0.0001.

The effect of various concentrations (2.5–200 μg/mL) of ALEE on human BCa cells for 24 h and 48 h and cytotoxicity and effect on proliferation were determined using tryphan blue assay and MTT, respectively (Figs. 4 and 5). Various ALEE concentrations used in the study are 2.5, 5 and 10 μg/mL, and they demonstrated no effect on the viability of both cells. Still, treatment with a concentration between 25 and 200 μg/mL revealed significant changes (P < 0.05). Treatment of MDA-MB 231 with 2.5, 5 and 10 μg/mL ALEE did not significantly alter cell viability compared to untreated cells (control). Similarly, treatment of MCF-7 with 2.5, 5 and 10 μg/mL ALEE did not show significant changes compared with the control (P > 0.05). Studies revealed in vitro anti-proliferative potential of Silicic acid, diethyl bis(trimethylsilyl) ester separated from *Lorabthus parasiticus* on breast cancer in a dose-dependent manner, which is in agreement with our findings^49^. (S)-(E)-(−)-4-Acetoxy-1-phenyl-2-dodecen-1-one (Quercetin) isolated from green tea revealed anti-proliferative effect against in PC-3 and LNCaP human prostate cancer cells^50^. Furthermore, studies revealed that quercetin isolated from plants inhibits proliferation, signal transduction and metastasis in cancer cell lines^51^.

**Figure 6:**
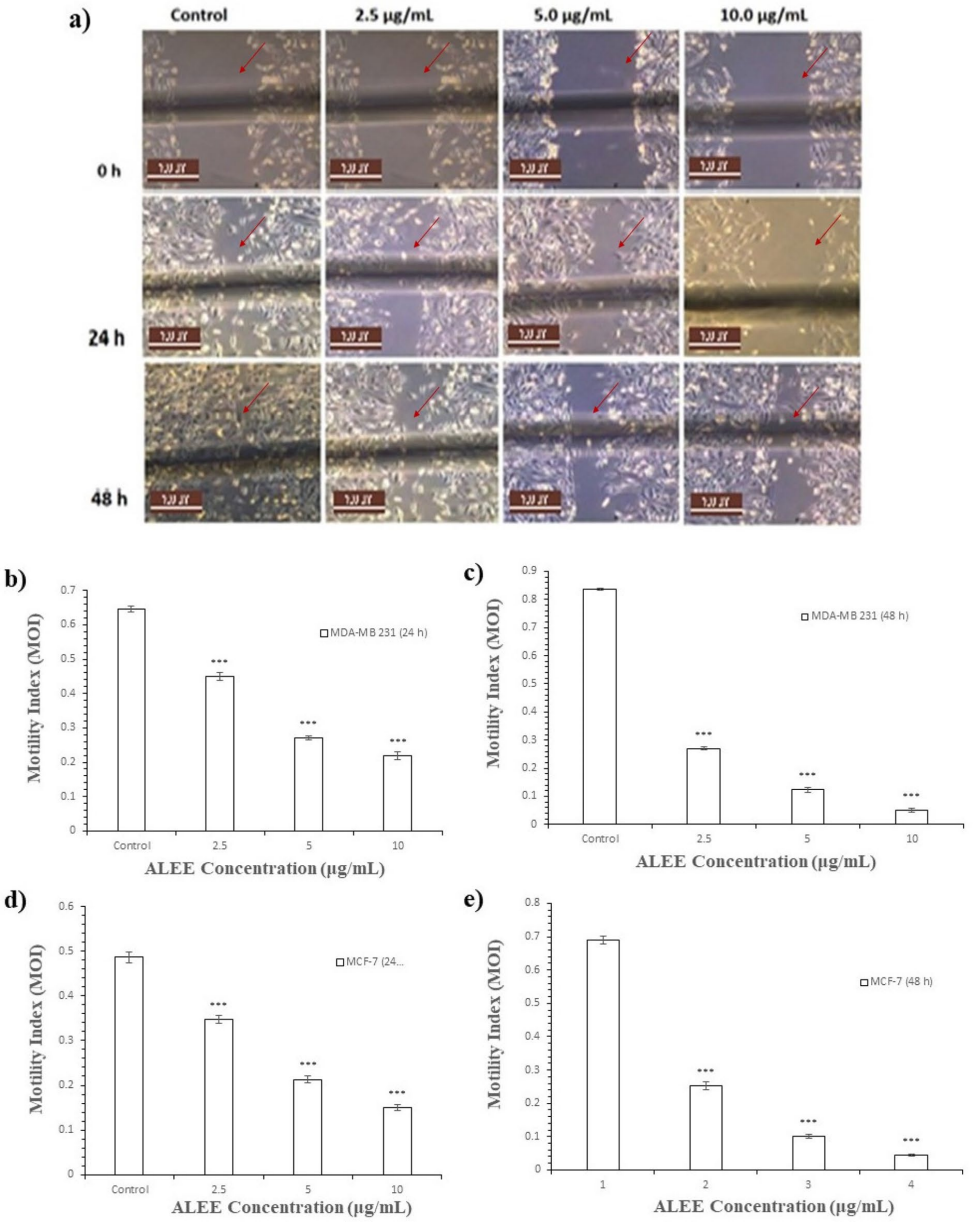
Effect of ALEE on lateral motility of MDA-MB 231 and MCF-7. (**a**) Typical phase-contrast light-microscopy (× 10) images obtained from wound healing assays of MDA-MB 231 cell following 24 h and 48 h incubation to show the wound. Scale bar (100 μm) applicable to all panels. Bar diagram showing motility index of MDAMB 231 cell following (**b**) 24 h incubation ± ALEE (**c**) 48 h incubation ± ALEE. Bar diagram showing motility index of MCF-7 cell following (**d**) 24 h incubation ± ALEE (**e**) 48 h incubation ± ALEE Data are presented as mean ± SEM of at least replicates experiments (*n* ≥ 3).

According to the study, the concentration of ALEE did not have a notable impact on the viability and growth of MDA-MB 231 and MCF-7 human BCa cells when compared to the control group. Nonetheless, the anti-migratory capacity of the cells was examined through the wound healing assay, and it was discovered that the lateral motility index (MOI) of MDA-MB 231 decreased with an increase in ALEE concentration and incubation duration. Figure 6 indicates that 10 μg/mL of ALEE had the most optimal motility index among the other concentrations. The MOI is more in MDA-MB 231 because the cells are metastatic and aggressive. In addition, all ALEE concentrations revealed significant differences relative to the control (P < 0.05) (Fig. 6). Similarly, the MCF-7 MOI was reduced with increased ALEE concentration and incubation period, as shown in Fig. 6d,e, and 10 μg/mL revealed the lowest and best MOI when compared with the remaining ALEE concentrations. MCF-7 is a less aggressive and weakly metastatic cell, which could be the reason for the lower MoI compared with MDA-MB 231 cells. The ALEE concentrations (2.5–10 μg/mL) revealed a decrease in the MOI of MCF-7 cells with increased concentrations and incubation time, and 10 μg/mL revealed more effect on lateral motility followed by 5 μg/mL (Fig. 6d,e; *n* ≥ 3). Nanoparticles synthesised using quercetin reached plant (*Ficus ingens*) revealed an effect on lateral motility of MDA-MB 231^13^. Medicinal plants containing quercetin as an active ingredient showed anti-metastatic activity on strongly and weakly metastatic MatLYLu and AT-2 rat prostate cancer cell models, respectively^52^.

### Anti-migratory potential prediction models

**Figure 7:**
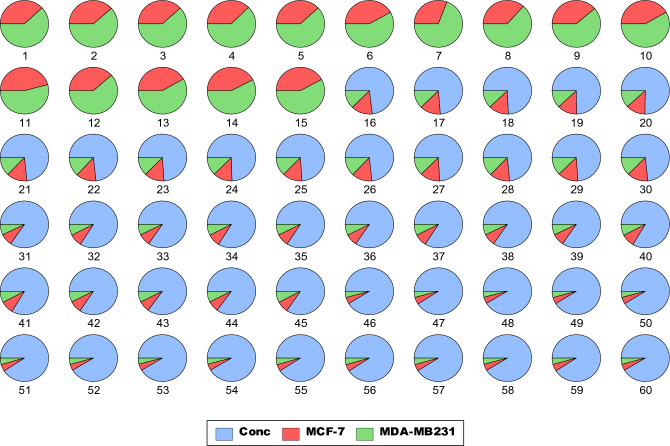
Visualized pie-chart distribution of our data set for both MDA-MB 231 and MCF-7 cells treated with various concentration of ALEE extract.

**Figure 8:**
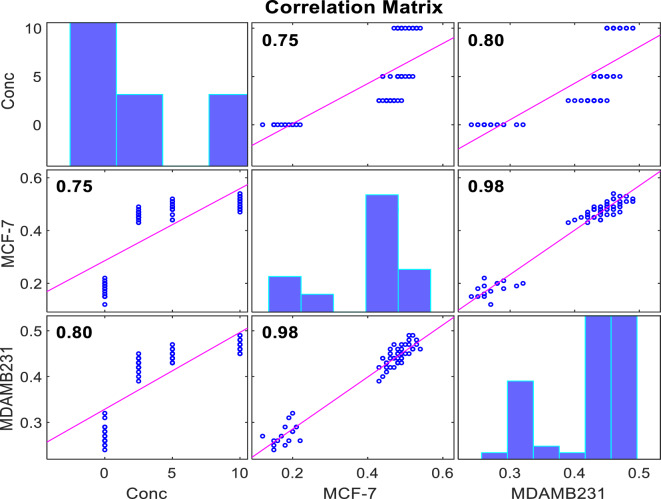
Correlation matrix between the raw data.

The AI-based models (MLP, XGB, and ELM) were analysed to predict in vitro cancer migration prediction in cells treated with ALEE based on our experimental data. Before the model calibration, statistical data analysis was conducted, as shown in Table 2. Generally, statistical analysis is done to understand the dataset. Furthermore, The AI-based models (MLP, XGB, and ELM) were analysed to predict in vitro cancer migration prediction in the MDA-MB 231 and MCF-7 human BCa, treated with ALEE based on our experimental data. The performance evaluation is checked by applying various criteria to compare the simulated and the observed values. The distribution between the different multiple parameters and the dataset used in the study was expressed as a visualised pie chart in Fig. 7, and the data set is well distributed. Furthermore, the correlation matrix shows the correlation between different parameters in a linear form. It can be seen from Fig. 8 that there is a high correlation between all the parameters, whereby the highest correlation in this study is between MDA-MB231 and MCF-7 having R-value = 0.98, and the lowest correlation exists between concentration and MCF-7 with R = 0.75. Similarly, the correlation matrix shows a robust correlation between all the variables and is in conformity with the correlation revealed by Adun et al.^53^.

**Table 2:** Descriptive statistic.

Variables	Mean	SE	Median	SD	Kurtosis	Skewness	Min	Max
Conc	4.375	0.48138	3.75	3.728753	− 1.14951	0.445876	0	10
MCF-7	0.405333	0.017451	0.47	0.135177	− 0.5607	− 1.10911	0.12	0.54
MDA-MB 231	0.402167	0.010146	0.435	0.078591	− 0.5463	− 1.04085	0.24	0.49

*Conc.* concentration, *SE* standard error of mean, *SD* standard deviation, *Min*. minimum, *Max.* maximum.

**Figure 9:**
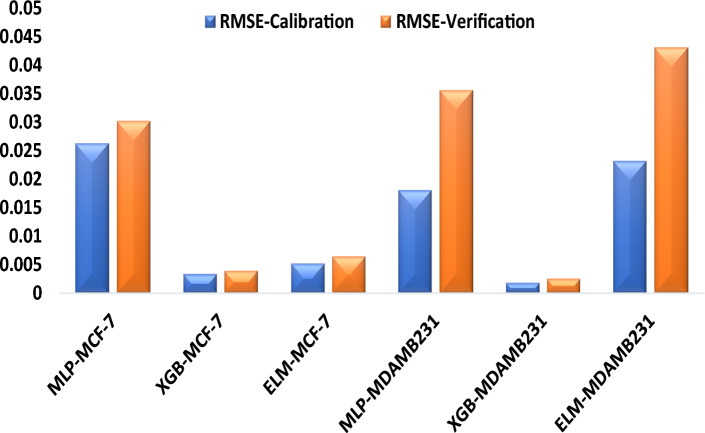
Relative mean square error (RMSE) for predicting MDA-MB 231 and MCF-7 human Breast Cancer treated with ALEE in both the calibration and verification stages.

**Figure 10:**
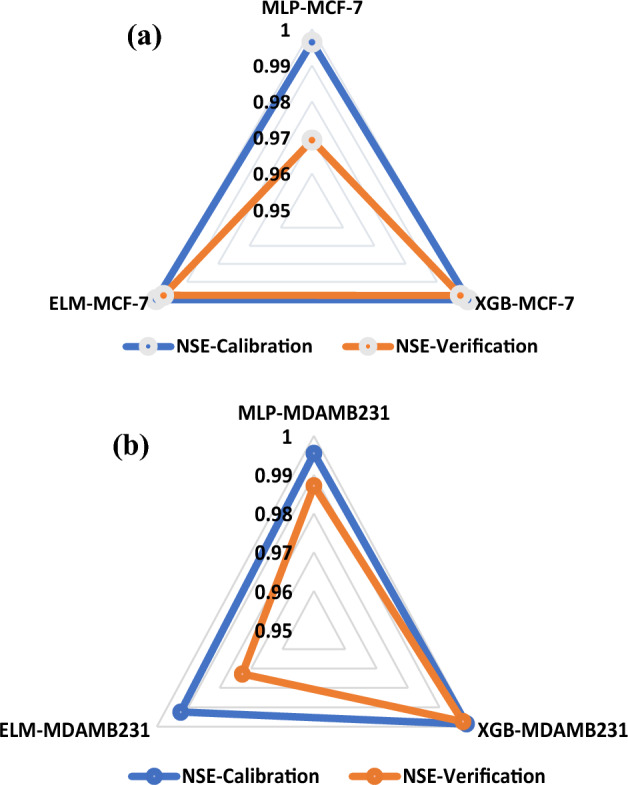
Radar chart for various variation determination and correlation coefficients (**a**) MCF-7 (**b**) MDA-MB 231.

The modelling performance of MLP, XGB, and ELM models, treated MDA-MB 231 and MCF-7, were compared to each other using RMSE and NSE, as shown in Table 2. Based on the predictive comparison of the models in Table 3, it can be shown clearly that all three data-driven models (MLP, XGB and ELM) can simulate the in vitro cancer migration potential prediction in the human BCa cells. XGB depicted the superiority over the other two non-linear models in the testing and training stages for modelling the performance of the cells. In regards to their error values, XGB shows the lowest RMSE values, XGB-MCF-7 = 0.0039 and XGB-MDAMB231 = 0.0025 in the testing phase, and the NSE as a goodness of fits which shows that XGB equally outperformed all the other AI-based models MLP and ELM and increase their performance efficiency up to 3% and 1% for MCF and 1% and 2% for MDA-MB231 respectively in the testing phase. The relative predictive accuracy regarding the relative error can also be demonstrated using a bar chart (Fig. 9), which reflects the performance of in vitro cancer metastasis prediction in human BCa cells in a surface radar chart showing the scale of NSE in the training and testing phases. It has been reported that the radar scale generally ranges between 0 and 1. The radar chart performance demonstrated that the versions in terms of NSE of treated BCa cell migration in highly and weakly metastatic human BCa cell lines follow the following order: XGB > ELM > MLP for MCF-7, and XGB > MLP > ELM for MDA-MB 231, respectively (Fig. 10). BCa subtypes were identified based on the immune signature in the tumour microenvironment for accurate assessment and treatment of BCa using the MLP model, and the study outcomes conform with our results^54^. The metastatic status of BCa and new therapeutic target provision were predicted using an efficient XGB model optimized by a grid search algorithm^55^. In addition, Benign or malignant types of BCa were classified using classification robustness ELM and based on input mammograms, and the outcomes are in agreement with our findings^56^. Similarly, the methanolic extract of *A. lebbeck* demonstrated good performance using other Al-based models^57^.

**Table 3:** Result of the ANN, ANFIS and MLR models.

	Calibration		Verification	
	NSE	RMSE	NSE	RMSE
MLP-MCF-7	0.9964	0.0263	0.9692	0.0302
XGB-MCF-7	0.9999	0.0033	0.9975	0.0039
ELM-MCF-7	0.9998	0.0052	0.9974	0.0064
MLP-MDA-MB231	0.9955	0.0181	0.9871	0.0356
XGB-MDA-MB231	0.9985	0.0018	0.9974	0.0025
ELM-MDA-MB231	0.9924	0.0232	0.9728	0.0432

## Conclusion

Our study has uncovered promising organic compounds in ALEE that possess medicinal properties, potentially aiding in the prevention of metastasis in human breast cancer. Interestingly, we observed that varied concentrations of the plant extract were non-toxic and had no impact on cell proliferation but displayed significant anti-migratory potential in both MDA-MB 231 and MCF-7 cells, with increasing concentration. Furthermore, we found that AI models, including MLP, XGB, and ELM, were effective in predicting the anti-migratory potential of ALEE. XGB demonstrated the highest performance efficiency, outperforming MLP and ELM models by 3% and 1% for MCF and 1% and 2% for MDA-MB231 during the testing phase. However, further studies are required to ascertain the anti-metastatic potential of the plant using various cell lines as well as to validate the anti-migratory potential of this plant, and additional computational models should be employed to improve performance.

## Data Availability

All data is included in the manuscript.
